# Microbial quotient responses to altitudinal and edaphic variability in Himalayan apple orchard soil

**DOI:** 10.3389/fmicb.2026.1957054

**Published:** 2026-10-08

**Authors:** Stanzin Khenrab, Sarabdeep Kour, Vikas Sharma, Tsering Lanzes, Tsewang Dolma, Disket Dolkar, Mohd Rafee, Sourav Singh, Manjot Singh Sidhu, Jigmet Namgial

**Affiliations:** 1 Division of Soil Science and Agricultural Chemistry, FoA, SKUAST-Jammu, Jammu, India; 2 Division of Fruit Science, FoH&F, SKUAST-Jammu, Jammu, India; 3 Division of Agricultural Extension Education, FoA, SKUAST-Jammu, Jammu, India; 4 Krishi Vigyan Kendra-Leh, Stakna, Ladakh, India; 5 Department of Commerce, University of Ladakh, Kargil, India; 6 Division of Silviculture and Agroforestry, FoH&F, SKUAST-Jammu, Jammu, India

**Keywords:** biological indicators, carbon-use efficiency, elevation gradient, enzyme activity, microbial biomass carbon, mountain horticulture, substrate normalisation, subsurface soil

## Abstract

**Introduction:**

Soil biological health is a critical determinant of sustainable fruit production in mountain ecosystems, yet microbial dynamics in high-altitude orchards remain poorly understood. Whereas total organic carbon (TOC) changes only slowly, the microbial quotient (MQ)—the ratio of microbial biomass carbon (MBC) to TOC—responds early and sensitively to shifts in organic matter turnover and soil biological quality.

**Methods:**

This study quantified MQ across four altitude classes (<1,550, 1,550–1,650, 1,650–1,850 and >1,850 m a.s.l.), three orchard age classes (<8, 8–14, and >14 years) and three soil depths (0–25, 25–50, and 50–75 cm) in apple orchards of the Jammu Himalaya, using 144 stratified samples. MBC and TOC were measured and MQ computed as (MBC/TOC) × 100; dehydrogenase (DHA) and alkaline phosphatase activities were determined as supporting indicators. Data were analysed by three-way ANOVA, Tukey HSD, Pearson correlation, principal component analysis (PCA) and regression.

**Results:**

MQ averaged 3.57 ± 0.08% (range 2.06–8.28%) and declined significantly with altitude, from 4.13% at <1,550 m to 3.11% at >1,850 m (*p* < 0.005), the inverse of the MBC and TOC trends. MQ was lowest in surface soil (2.82%) and highest in the 25–50 cm layer (4.14%; *p* < 0.005) and decreased modestly with orchard age (*p* = 0.010); significant altitude × depth and altitude × age interactions were detected. MQ correlated strongly and negatively with TOC (*r* = −0.745; *R*^2^ = 0.556) but was decoupled from MBC (*r* = −0.082, ns), indicating that MQ carries information about the substrate-normalised size of the biomass pool that absolute MBC does not. PCA separated a biomass-substrate axis (PC1, 48.3%) from an MQ-dominated axis.

**Discussion:**

Because microbial carbon-use efficiency, community composition and necromass were not measured directly, the efficiency interpretation of these gradients is advanced as a hypothesis consistent with the data rather than as a demonstrated mechanism. The study nevertheless establishes MQ as a sensitive, substrate-normalised descriptor of biological soil quality and provides the first MQ baseline for mountain apple orchards of the north-western Himalaya.

## Graphical abstract

**Figure:**
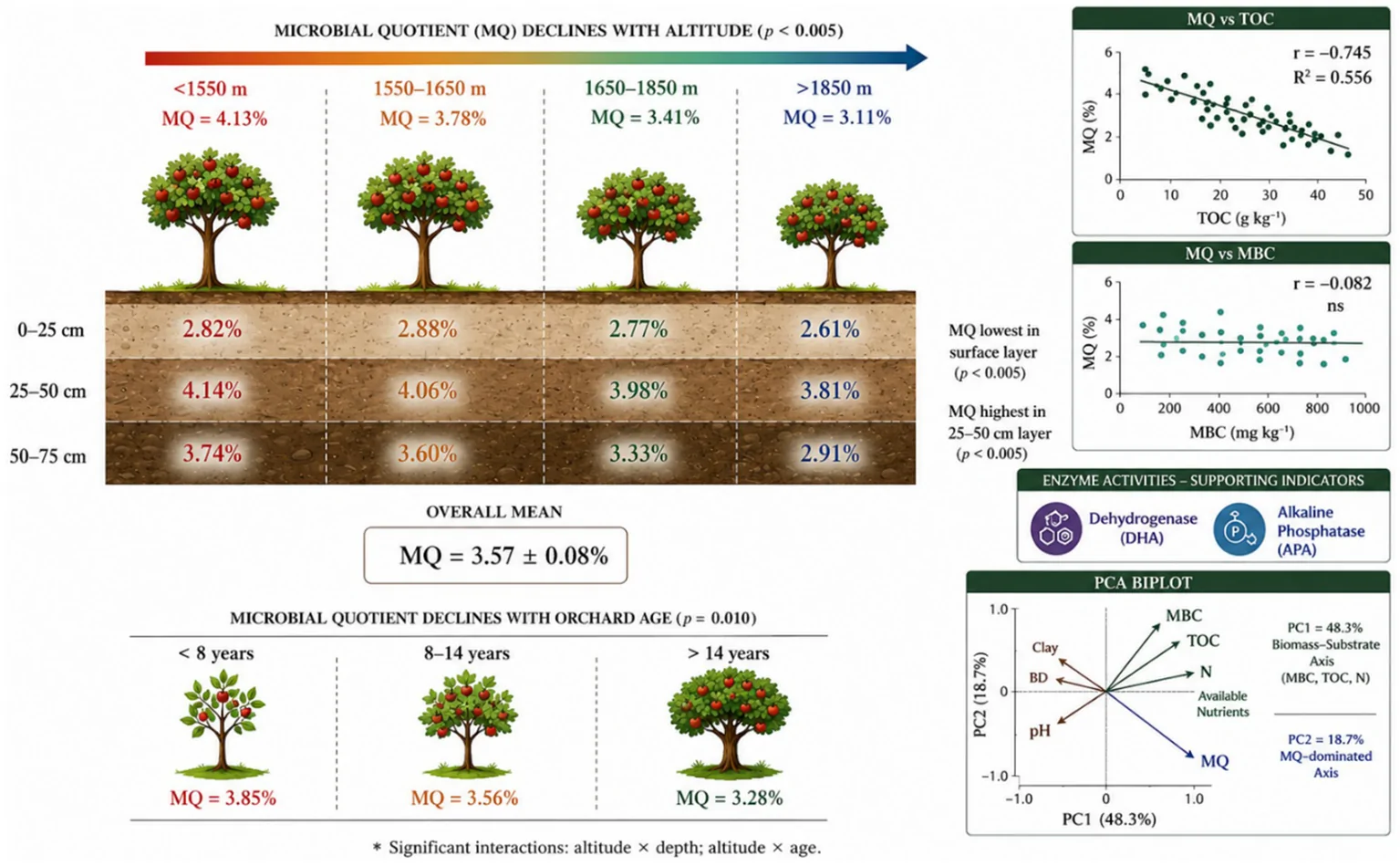

## 1 Introduction

Soil health underpins the capacity of agricultural ecosystems to cycle nutrients, stabilise organic matter and sustain productivity over the long term (Doran and Zeiss, 2000; Lehmann et al., 2020). The concept integrates physical, chemical and biological attributes, but these differ greatly in the speed with which they respond to management. Physical and chemical attributes change slowly, whereas the biological component reacts rapidly to management and environmental perturbation, making microbial parameters early and sensitive sentinels of change (Schloter et al., 2018; Nannipieri et al., 2017). In perennial systems such as apple orchards, where the soil is rarely disturbed and carbon inputs are dominated by root turnover and leaf litter, biological indicators offer a particularly informative window on belowground functioning (Bowles et al., 2014; Lori et al., 2017).

The impetus to monitor soil biological health has intensified as agriculture confronts the twin pressures of sustaining productivity and mitigating climate change (Lehmann et al., 2020; Bardgett and van der Putten, 2014). Microbial communities govern the balance between carbon mineralisation and stabilisation. Small shifts in the efficiency with which they process carbon therefore scale up to large consequences for the terrestrial carbon balance (Schimel, 2018; Wieder et al., 2013; Bradford et al., 2016; Bhattacharyya et al., 2022). Contemporary soil microbiome ecology has reinforced this view, showing that community composition, life-history strategy and network structure—not biomass alone—determine how organic inputs are partitioned between respiration and stabilised pools (Singh et al., 2020; Yang et al., 2023; Hou et al., 2026). Single measurements of biomass or activity can be misleading when soils differ in organic matter content, so integrative microbial indices are increasingly built into soil-health frameworks; the microbial quotient answers this need by placing biomass in the context of the substrate that supports it (Bunemann et al., 2018; Anderson, 2003).

Microbial biomass carbon (MBC) represents the living, metabolically active fraction of soil organic matter and, although it typically constitutes only 1–5% of total organic carbon (TOC), it mediates most organic matter transformations (Jenkinson and Powlson, 1976; Wardle, 1992). Because it turns over far more rapidly than bulk organic matter, changes in MBC frequently precede detectable shifts in TOC (Powlson et al., 1987) and the biomass is increasingly recognised not merely as an agent of decomposition but as the principal precursor of stable, mineral-associated organic matter through microbial necromass (Kallenbach et al., 2016; Wang et al., 2021). The microbial quotient (MQ), defined as the ratio of MBC to TOC and expressed as a percentage, normalises microbial biomass to the size of the organic substrate pool (Sparling, 1992; Anderson, 2003). This normalisation converts an abundance measure into a relative measure, indicating what proportion of the available carbon the community holds as living biomass; it is therefore widely interpreted as an index of substrate quality, microbial carbon-use efficiency and the direction of organic matter accumulation (Anderson and Domsch, 1989; Anderson and Domsch, 2010). A declining MQ is generally read as evidence that carbon is accumulating faster than the community can assimilate it, or that substrate quality, pH or climatic stress is constraining efficiency, whereas a rising MQ is taken to reflect efficient incorporation of fresh substrate (Insam and Domsch, 1988; Manzoni et al., 2012). Being dimensionless and substrate-normalised, MQ permits meaningful comparison across soils differing widely in organic carbon content, a property especially valuable along steep environmental gradients (Raiesi and Beheshti, 2015; Marinari et al., 2022).

Supporting enzymatic indicators strengthen the biological interpretation of MQ. Dehydrogenase activity (DHA) reflects the oxidative metabolic activity of viable cells and serves as a proxy for overall microbial activity (Moscatelli et al., 2018), while alkaline phosphatase mediates organic phosphorus mineralisation and indexes microbial nutrient acquisition (Tabatabai and Bremner, 1969; Sinsabaugh et al., 2008). Eco-enzymatic stoichiometry holds that communities allocate resources to enzyme production in proportion to the nutrients limiting growth, so activity patterns reveal the resource economy of the soil (Cui et al., 2018). The same logic extends to the soil itself: ecological stoichiometry of carbon, nitrogen and phosphorus (C:N:P) constrains microbial biomass, nutrient cycling and biological efficiency and stoichiometric imbalance between resource supply and microbial demand is now recognised as a fundamental regulator of how much assimilated carbon is retained as biomass (Liu et al., 2023; Sinsabaugh et al., 2016). Interpreted alongside MQ, enzyme activities therefore indicate not only how much carbon and nutrient processing occurs but the resource context in which the resident community operates—a functional dimension that a biomass measurement alone cannot supply (German et al., 2011).

Three environmental gradients exert particularly strong control on these processes in mountain orchards: altitude, stand age and soil depth. Declining temperatures with elevation slow metabolism and decomposition, allowing organic carbon to accumulate while altering moisture, vegetation inputs and soil reaction (Margesin et al., 2009; Nottingham et al., 2019). The net effect on the biomass-to-carbon ratio is not straightforward: lower temperatures generally depress carbon-use efficiency through higher maintenance respiration, yet the accompanying build-up of substrate can dilute biomass relative to TOC (Frey et al., 2013; Feng et al., 2021). Reports from the Himalaya and other mountain systems consistently show absolute TOC rising with elevation, yet the behaviour of the MBC-to-TOC ratio is far less documented (Tashi et al., 2016; Bangroo et al., 2017). Orchard age adds a second axis: young orchards supply less but fresher, more labile substrate, whereas mature orchards build larger but increasingly stabilised carbon stocks and receive a longer history of fertiliser inputs, so MQ can decline with age even as absolute MBC rises (Six et al., 2006; Insam and Domsch, 1988; Zheng et al., 2024). Soil depth provides a third: surface horizons receive the bulk of fresh inputs and hold the largest biomass, enzyme activity and TOC, so the depth behaviour of MQ depends on the relative rates at which biomass and substrate decline down the profile and subsurface layers where substrate is scarcer can paradoxically support a higher quotient if the residual carbon is preferentially associated with active microbial fractions (Loeppmann et al., 2016; Rasmussen et al., 2018).

The north-western Himalaya is a globally important temperate fruit-growing region, with apple as its flagship crop (Kumar et al., 2018; Khan and Hanjra, 2023). The Jammu division was selected for this study for three specific reasons rather than simply as a temperate fruit-growing region. First, its apple belt spans an unusually compressed altitudinal range, from below 1,550 m to above 1,850 m a.s.l. within a single administrative division, so that a steep climatic gradient can be sampled while parent material, cultivar spectrum and cultural practice remain broadly comparable. Second, orchards of contrasting establishment age occur side by side within each altitudinal band, allowing altitude and stand age to be crossed factorially in the field. Third, in contrast to the well-characterised Kashmir and Himachal belts, the soils of the Jammu apple belt have never been assessed for biological quality, so no biological benchmark exists for a region where orchard area is expanding rapidly onto fragile mountain soils (Pareek, 2017; Dad and Abdollahi, 2021). Most Indian Himalayan studies of apple orchards have concentrated on fertility, nutrient management and, more recently, organic carbon stocks, while the biological dimension has received comparatively little attention; the few biological investigations rarely report MQ explicitly and almost none resolve it simultaneously across altitude, age and depth within a single factorial design (Sharma et al., 2021; Marinari et al., 2022; Jakhro et al., 2025b). This gap matters because management recommendations are increasingly framed around carbon sequestration, yet without a substrate-normalised biological metric it is impossible to judge whether accumulating carbon is being processed by an active community or is simply accruing in a biologically inert form (Doran and Zeiss, 2000; Singh et al., 2020).

Against this background, the present study quantifies MQ and its supporting biological indicators across the principal environmental gradients of Himalayan apple orchards. The specific objectives were to: (i) quantify the microbial quotient across four altitudinal classes; (ii) determine the influence of orchard age on MQ; (iii) assess the depth-wise distribution of MQ; (iv) investigate the relationships between MQ and the supporting biological indicators MBC, TOC, DHA and alkaline phosphatase; and (v) evaluate the usefulness of MQ as an indicator of biological soil health in mountain apple production systems. We hypothesised that MQ would diverge from and in places oppose, the trends in absolute MBC and TOC, thereby demonstrating its distinct diagnostic value as a substrate-normalised measure. The novelty of this work is threefold. It provides the first factorial resolution of MQ across altitude, orchard age and soil depth simultaneously in any apple production system, rather than along a single gradient as in previous elevation or chronosequence studies. It reports the first quantitative MQ benchmarks for the Jammu Himalayan apple belt, a region for which no biological soil-quality baseline previously existed. And it demonstrates statistically—through the contrast between the MQ-TOC and MQ-MBC relationships and through the emergence of a dedicated MQ axis in ordination space—that MQ is not redundant with the absolute biomass and carbon pools that dominate existing soil-health batteries. These are, by design, baseline observations: the study characterises the pattern and identifies its proximate statistical driver and the mechanisms proposed to explain that pattern are advanced as hypotheses for subsequent testing.

## 2 Materials and methods

### 2.1 Study area

The study was conducted in established apple orchards of the Jammu Himalayan region, north-western India, which lies approximately between 32°40′–33°50′N latitude and 74°30′–75°40′E longitude. Sampling spanned four representative apple-growing locations distributed across the altitudinal belt of the division—Bani (Kathua district), Bhaderwah (Doda district), Gulabgarh (Reasi district) and Kishtwar (Kishtwar district). The region experiences a temperate sub-humid climate, with annual mean temperatures decreasing and precipitation regimes shifting along the elevation gradient; lower-altitude sites are warmer, while higher-altitude sites such as Kishtwar and Gulabgarh experience markedly cooler conditions. The soils are predominantly developed on mountain parent material under temperate orchard vegetation. The geographic distribution of the sampling locations is shown in Figure 1.

**Figure 1:**
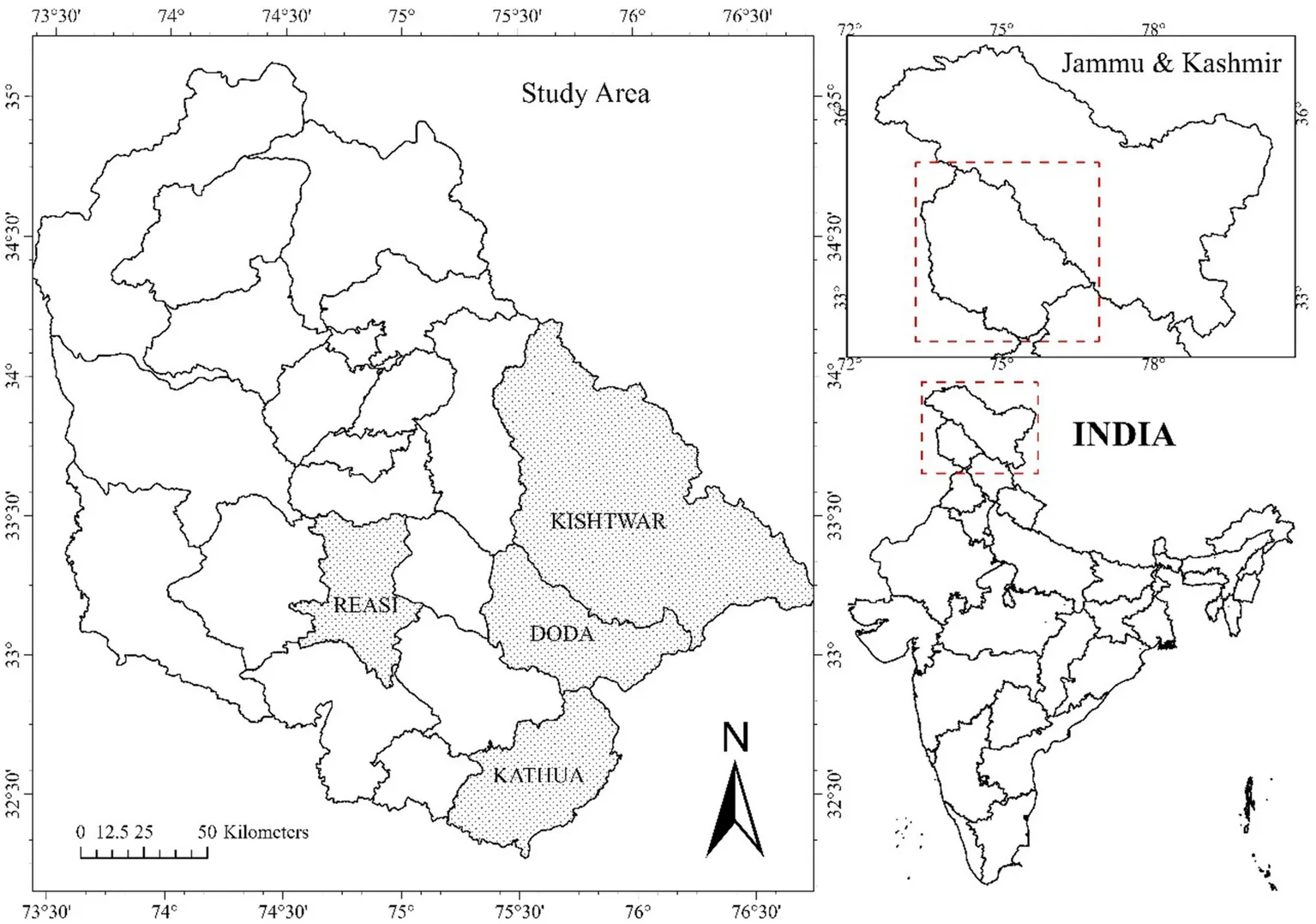
Study area map showing the selected apple-growing districts of the Jammu Himalayan region, India.

### 2.2 Experimental design and sampling

A stratified sampling design was adopted to capture biological variability across three crossed factors. Four altitude classes were delineated: <1,550 m (A1), 1,550–1,650 m (A2), 1,650–1,850 m (A3) and >1,850 m a.s.l. (A4). Orchards were grouped into three age classes: <8 years (young), 8–14 years (intermediate) and >14 years (mature). Within each orchard, soil was sampled at three depths: 0–25 cm (D1, surface), 25–50 cm (D2, subsurface) and 50–75 cm (D3, deep subsurface). The complete factorial combination of four altitudes, three age classes and three depths, with four replicate orchards per combination, yielded 144 soil samples.

The four replicates per altitude × age combination were four separate, spatially independent orchards under different ownership, not four sampling points within a single orchard; each replicate orchard contributed one composite sample per depth, so that the replicate is the orchard and pseudoreplication within orchards was avoided. Replicate orchards belonging to the same combination were separated by at least 4 meters. Orchards were further selected for uniformity of management so that the altitude, age and depth effects would not be confounded by husbandry. All selected orchards were rainfed with supplemental protective irrigation only and were under the same dominant cultivar group. All selected orchards received annual winter pruning of comparable intensity and followed the regionally recommended fertiliser schedule, consisting of farmyard manure (FYM) supplemented with inorganic N, P and K. Annual application rates generally ranged from approximately 30–100 kg FYM, 350–700 g N, 175–500 g P_2_O_5_ and 350–1,000 g K_2_O per tree, with variation across orchards. None of the selected orchards had received lime, biochar or other soil amendments and none had been subject to inter-row cultivation, cover cropping or herbicide-based clean cultivation that would alter carbon inputs differentially. Orchards were additionally matched for uniformity in vigour and planting geometry and their locations were georeferenced using a handheld GPS receiver to ensure spatial accuracy and reproducibility. Eroded knolls, depressions, saline patches and fence lines were avoided during collection. Composite samples were drawn from each depth, air-dried, gently ground and passed through a 2 mm sieve; a field-moist sub-sample was stored at 4 °C for the determination of microbial biomass carbon and enzyme activities.

### 2.3 Laboratory analyses

Total organic carbon (TOC) was determined by wet oxidation following the principle of Walkley and Black (1934) and expressed on an oven-dry basis (g kg^−1^). MBC was measured by the chloroform fumigation-extraction method (Vance et al., 1987; Brookes et al., 1985), with biomass carbon calculated from the difference in 0.5 M K_2_SO_4_-extractable carbon between fumigated and non-fumigated sub-samples and divided by an extraction efficiency factor (k_EC_) of 0.45 (Wu et al., 1990); MBC is reported in μg g^−1^. Soil reaction (pH) was measured in a 1:2.5 soil-water suspension and bulk density (BD) by the core method (Van Reeuwijk, 2002).

DHA was assayed by the reduction of 2,3,5-triphenyl tetrazolium chloride to triphenyl formazan (TPF) following Casida et al. (1964); field-moist soil was incubated with substrate in the dark at 37 °C for 24 h, the formazan produced was extracted in methanol and quantified colorimetrically at 485 nm and activity is expressed as μg TPF g^−1^ h^−1^. Alkaline phosphatase activity was determined by the release of p-nitrophenol (PNP) from p-nitrophenyl phosphate after incubation for 1 h at 37 °C in modified universal buffer adjusted to pH 11.0 (Tabatabai and Bremner, 1969), with the released PNP measured at 400 nm and activity expressed as μg PNP g^−1^ h^−1^. All determinations were performed on each of the 144 samples and analytical duplicates were run for every sample with re-analysis where the duplicate deviation exceeded 5%.

### 2.4 Calculation of the microbial quotient

The microbial quotient (MQ) was computed for every sample as the ratio of microbial biomass carbon to total organic carbon, expressed as a percentage (Sparling, 1992; Anderson, 2003):

MQ(%)=(MBC/TOC)×100

Because MBC was expressed in μg g^−1^ (equivalent to mg kg^−1^) and TOC in g kg^−1^, both were placed on a common mass basis before computation, so that MQ correctly represents the proportion of total organic carbon held in the living microbial biomass.

### 2.5 Statistical analysis

Descriptive statistics (mean, standard error, standard deviation, coefficient of variation and range) were computed for all biological indicators. The effects of altitude, orchard age and soil depth on MQ were tested by three-way analysis of variance (ANOVA) including all two-way and the three-way interaction and means were separated by Tukey’s honestly significant difference (HSD) test at *p* = 0.05, with compact letter displays indicating significant groupings. Before ANOVA, the underlying assumptions were verified: normality of model residuals was assessed by the Shapiro–Wilk test together with visual inspection of quantile-quantile plots and homogeneity of variance across factor-level combinations was tested by Levene’s test. Pearson correlation analysis quantified bivariate associations among MQ, MBC, TOC, DHA, alkaline phosphatase, pH and BD. Principal component analysis (PCA) was performed on the standardised biological indicators to identify the principal axes of variation and the position of MQ within the indicator space.

All analyses were conducted in Python using pandas and NumPy for data handling, SciPy for descriptive statistics, Levene and Pearson correlation, SPSS for the factorial ANOVA, Tukey HSD and principal component analysis. Figures were produced with Matplotlib and seaborn and rendered at 300 dpi.

## 3 Results

### 3.1 Descriptive statistics of biological indicators

Across the 144 orchard soil samples, MQ averaged 3.57 ± 0.08% and ranged from 2.06 to 8.28%, with a moderate coefficient of variation of 28.4% (Table 1). MBC averaged 817.3 ± 14.2 μg g^−1^ and TOC 24.6 ± 0.7 g kg^−1^; TOC was the most variable carbon indicator (CV 32.7%). DHA and alkaline phosphatase activities averaged 108.4 ± 1.2 and 25.7 ± 0.8 units, respectively. Alkaline phosphatase was the most variable indicator in the dataset (CV 37.1%), exceeding the variability of both TOC and MQ. This high dispersion is expected for a phosphorus-acquisition enzyme, whose activity responds sharply to local phosphorus availability, pH and the intensity of phosphatic fertiliser use, all of which differ appreciably among individual orchards; the corresponding correlations and regression coefficients involving alkaline phosphatase should therefore be interpreted with this greater dispersion in mind. Soil pH (mean 6.89) was mildly acidic to neutral and bulk density averaged 1.33 g cm^−3^, both with low variability (CV < 8%). The mean MQ fell within the 1–5% range commonly reported for cultivated and orchard soils (Anderson and Domsch, 1989; Sparling, 1992) and the moderate variability of MQ relative to the more variable TOC pool is consistent with earlier observations that the ratio behaves as a more stable integrative index than its component measures (Anderson, 2003; Raiesi and Beheshti, 2015).

**Table 1:** Descriptive statistics of microbial quotient and supporting soil biological indicators in Himalayan apple orchard soils (*n* = 144).

Indicator (unit)	Minimum	Maximum	Mean	SE	SD	CV (%)
Microbial quotient (%)	2.06	8.28	3.57	0.08	1.01	28.42
Microbial biomass C (μg g^−1^)	526.10	1207.90	817.32	14.17	169.98	20.80
Total organic C (g kg^−1^)	8.90	40.80	24.62	0.67	8.04	32.65
Dehydrogenase activity (μg TPF g^−1^ h^−1^)	70.20	134.90	108.39	1.24	14.91	13.75
Alkaline phosphatase (μg PNP g^−1^ h^−1^)	8.70	46.60	25.66	0.79	9.52	37.10
Soil reaction (pH)	5.73	8.10	6.89	0.04	0.53	7.71
Bulk density (g cm^−3^)	1.07	1.60	1.33	0.01	0.10	7.79

SE, standard error; SD, standard deviation; CV, coefficient of variation. TPF, triphenyl formazan; PNP, p-nitrophenol.

### 3.2 Three-way analysis of variance

Three-way ANOVA showed that MQ was significantly affected by all three main factors (Table 2). Soil depth exerted the strongest control (*F* = 68.1, *p* < 0.005), followed by altitude (*F* = 22.8, *p* < 0.005) and orchard age (*F* = 4.8, *p* = 0.010). The altitude × depth interaction was highly significant (*F* = 14.6, *p* < 0.005) and the altitude × age interaction was significant (*F* = 3.2, *p* = 0.007), as was the three-way interaction (*F* = 1.9, *p* = 0.045); the age × depth interaction was not significant (*p* = 0.194). The model explained 76.4% of the variation in MQ. The dominant influence of soil depth and altitude over orchard age echoes reports that vertical and climatic gradients structure microbial-to-carbon ratios more strongly than stand age in perennial systems (Fierer et al., 2009; Feng et al., 2021).

**Table 2:** Three-way analysis of variance for the microbial quotient (MQ) as affected by altitude, orchard age and soil depth.

Source of variation	df	Sum of squares	*F*-value	*p*-value
Altitude (A)	3	21.93	22.78	<0.005
Orchard age (B)	2	3.07	4.78	0.010
Soil depth (C)	2	43.70	68.10	<0.005
A × B	6	6.09	3.17	0.007
A × C	6	28.18	14.64	<0.005
B × C	4	1.98	1.55	0.194
A × B × C	12	7.23	1.88	0.045
Residual	108	34.65	–	–

Significant effects (*p* < 0.05) are indicated by the corresponding *p*-values; the model coefficient of determination was *R*^2^ = 0.764.

### 3.3 Altitudinal variation in microbial quotient

MQ declined consistently and significantly with increasing altitude (Table 3, Figure 2). The highest mean MQ occurred at the lowest altitude class, <1,550 m (4.13%) and the lowest at >1,850 m (3.11%), a reduction of approximately 25% across the gradient. Tukey HSD grouped the two lower-altitude classes (<1,550 m, 4.13%; 1,550–1,650 m, 3.71%) together and significantly above the two higher classes (1,650–1,850 m, 3.32%; >1,850 m, 3.11%). This downward trend in MQ is the inverse of the well-documented increase in absolute MBC and TOC with elevation, showing that the proportion of organic carbon held as living microbial biomass falls as substrate accumulates at higher, cooler sites. A comparable decline in microbial metabolic efficiency along elevation gradients, despite rising absolute carbon, has been reported for other mountain soils (Feng et al., 2021).

**Table 3:** Altitude-wise mean microbial quotient (MQ, %) with standard error and Tukey HSD groupings in Himalayan apple orchard soils.

Altitude class	*n*	Mean MQ (%)	SE	Tukey group
<1,550 m	36	4.13	0.24	a
1,550–1,650 m	36	3.71	0.14	a
1,650–1,850 m	36	3.32	0.12	b
>1,850 m	36	3.11	0.09	b

Means followed by a common letter are not significantly different at *p* = 0.05 (Tukey HSD).

**Figure 2:**
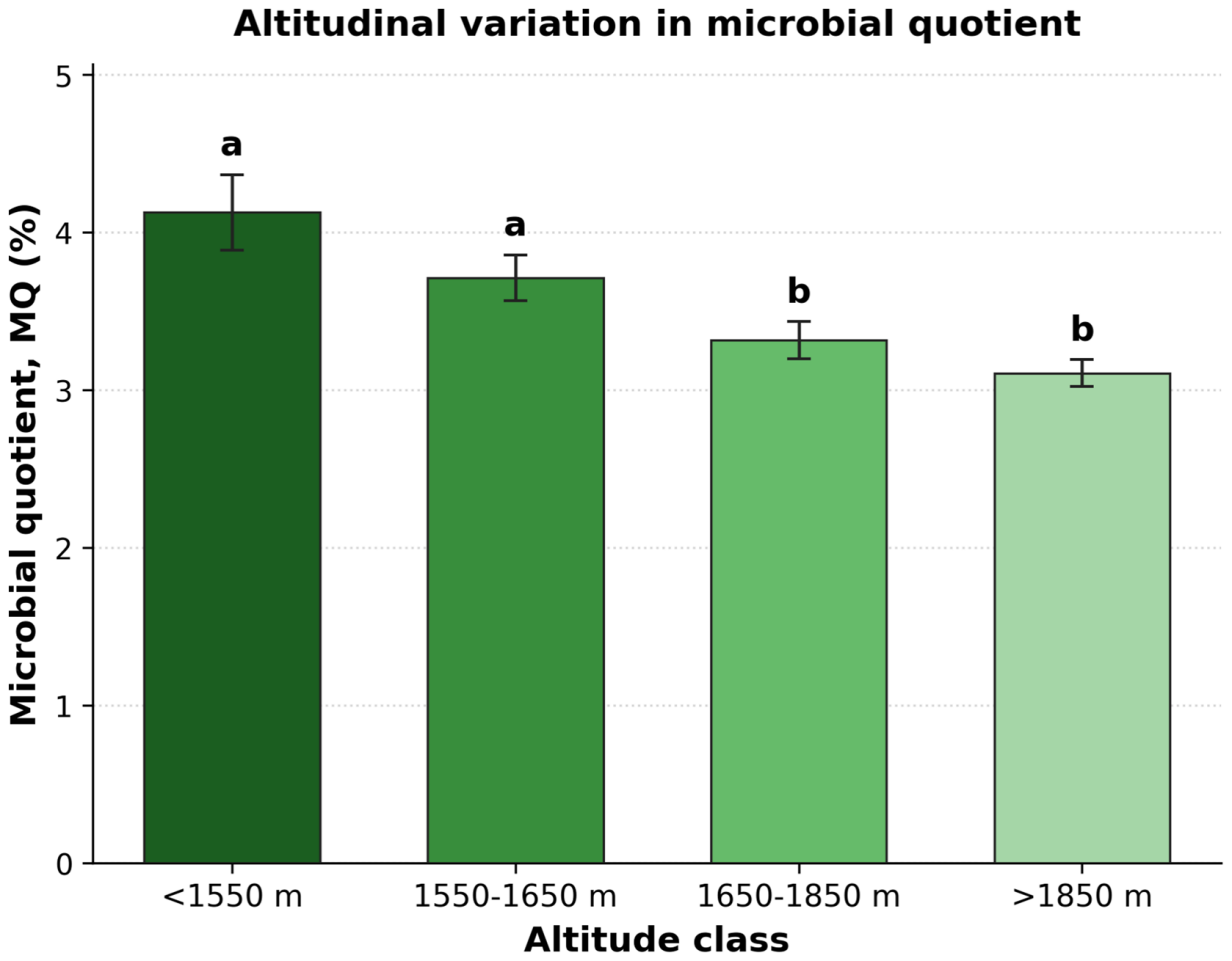
Altitudinal variation in the microbial quotient (MQ) of Himalayan apple orchard soils. Bars are means (*n* = 36 per class) ± standard error; different letters denote significant differences (Tukey HSD, *p* < 0.05).

### 3.4 Orchard age effects on microbial quotient

Although the ANOVA identified a significant overall effect of orchard age (*p* = 0.010), the magnitude of the age effect was modest and the pairwise Tukey separations were not significant (Table 4, Figure 3). Mean MQ was highest in young orchards (<8 years, 3.77%) and declined to 3.48% in intermediate (8–14 years) and 3.45% in mature (>14 years) orchards. The pattern is consistent with a progressive shift from a substrate-limited condition in young orchards toward a more carbon-rich condition as orchards mature and accumulate stabilised organic matter, a trajectory reported in chronosequence studies of perennial plantations (Six et al., 2006; Zhang et al., 2019).

**Table 4:** Orchard age-wise mean microbial quotient (MQ, %) with standard error and Tukey HSD groupings.

Orchard age class	*n*	Mean MQ (%)	SE	Tukey group
<8 yr	48	3.77	0.18	a
8–14 yr	48	3.48	0.13	a
>14 yr	48	3.45	0.12	a

Means followed by a common letter are not significantly different at *p* = 0.05 (Tukey HSD); the overall age effect was significant in the ANOVA (*p* = 0.010).

**Figure 3:**
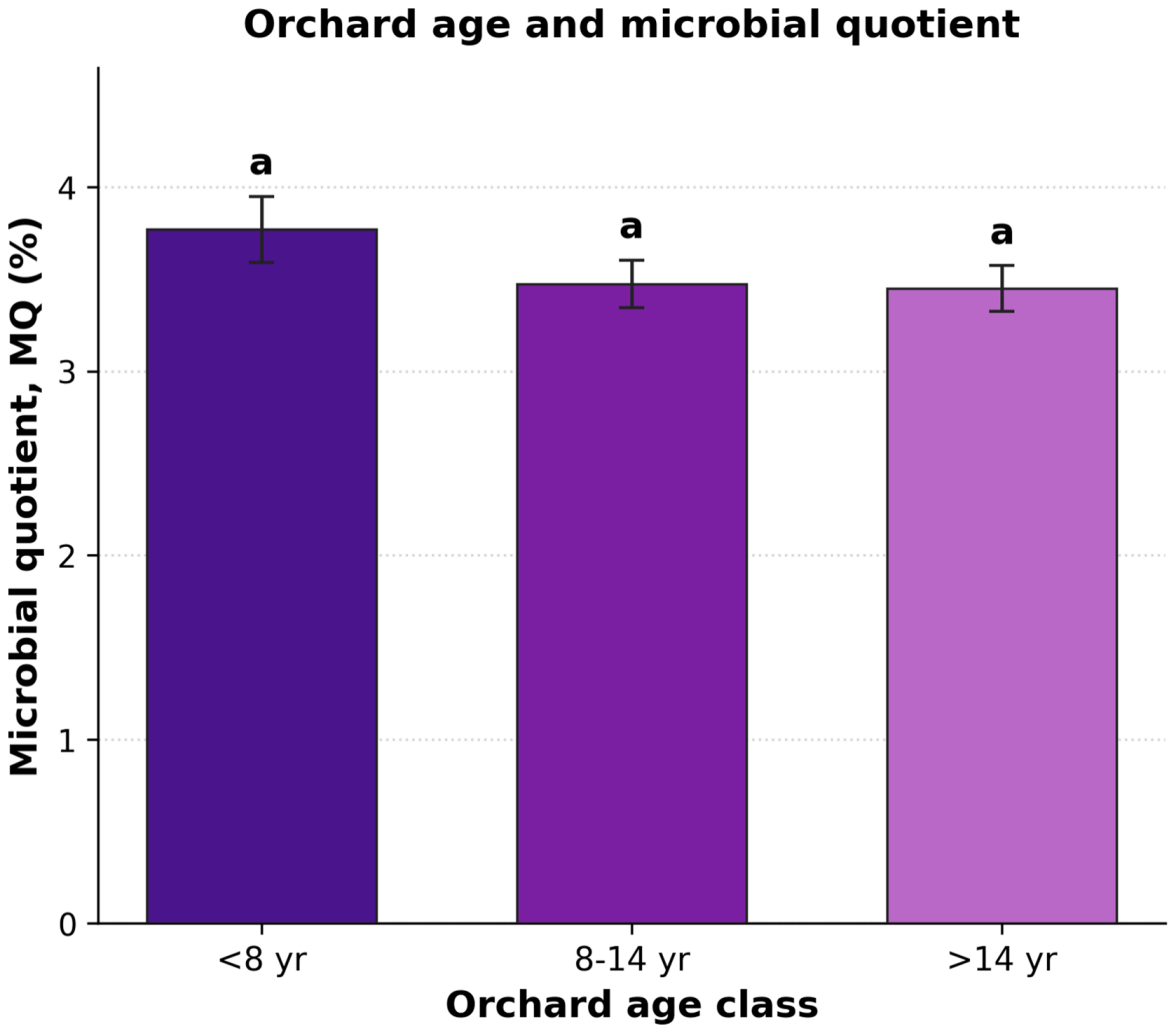
Variation in the microbial quotient (MQ) of apple orchard soils across three orchard age classes (means ± SE, *n* = 48 per class).

### 3.5 Depth-wise distribution of microbial quotient

MQ displayed a pronounced and statistically robust depth pattern but—contrary to the monotonic decline shown by absolute MBC and TOC—was lowest at the surface and peaked in the subsurface (Table 5, Figure 4). Surface soil (0–25 cm) recorded the lowest MQ (2.82%), which rose significantly to a maximum of 4.14% in the 25–50 cm layer and remained elevated at 3.73% in the 50–75 cm layer. Tukey HSD separated the surface layer (group b) from the two subsurface layers (group a). This subsurface maximum shows that, although the surface horizon contains the most carbon and the largest microbial biomass, a greater proportion of the residual carbon at depth is held in the microbial pool. Comparable subsurface enrichment of microbially associated carbon has been documented in profile-resolved studies (Rasmussen et al., 2018; Loeppmann et al., 2016; Jakhro et al., 2025a).

**Table 5:** Depth-wise mean microbial quotient (MQ, %) with standard error and Tukey HSD groupings.

Soil depth	*n*	Mean MQ (%)	SE	Tukey group
0–25 cm	48	2.82	0.07	b
25–50 cm	48	4.14	0.12	a
50–75 cm	48	3.73	0.17	a

Means followed by a common letter are not significantly different at *p* = 0.05 (Tukey HSD).

**Figure 4:**
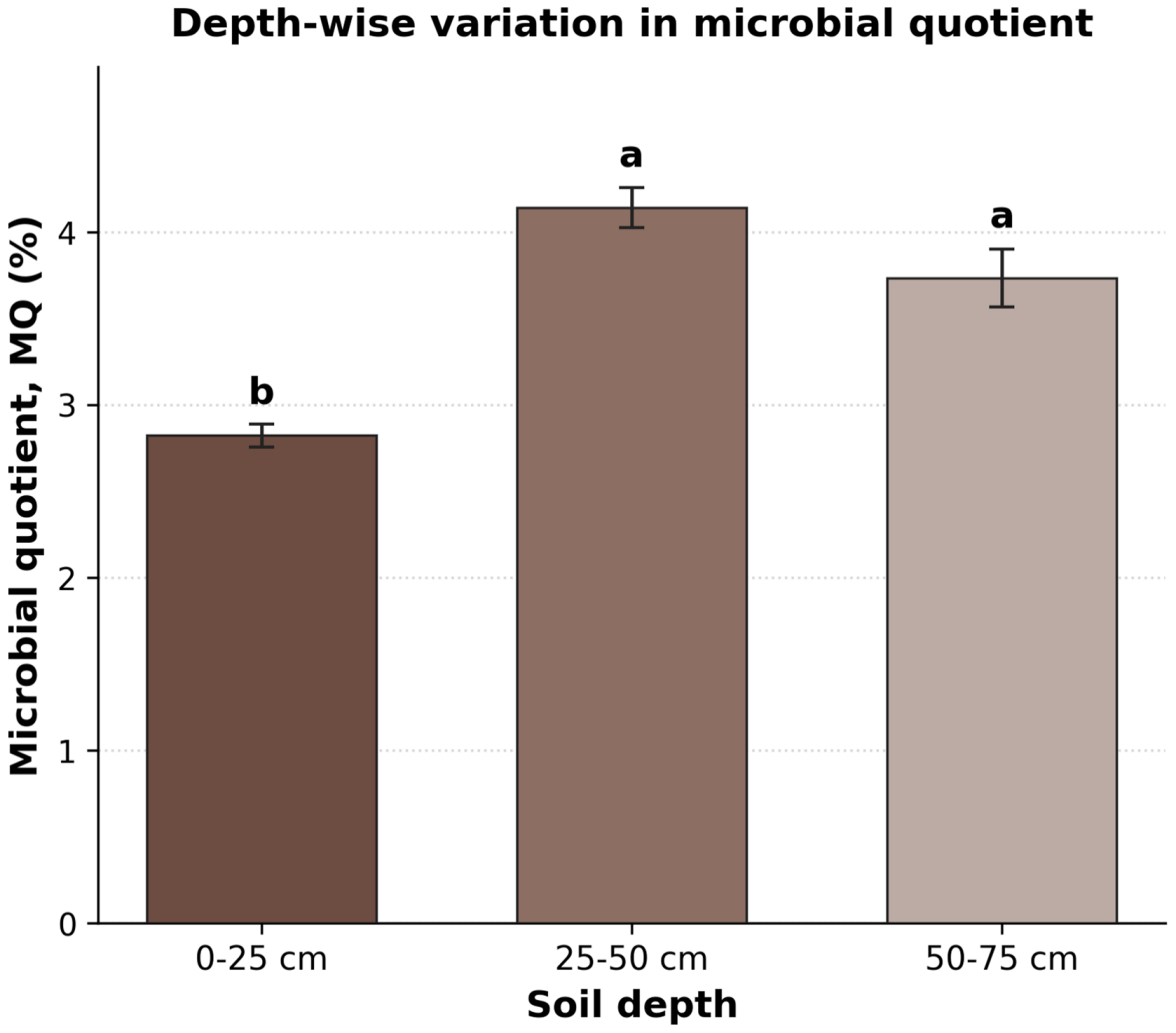
Depth-wise variation in the microbial quotient (MQ) of apple orchard soils (means ± SE, *n* = 48 per depth).

### 3.6 Altitude × orchard age interaction

The significant altitude × age interaction is visualised as a heatmap of mean MQ in Figure 5. The altitudinal decline in MQ was steepest in young orchards, where MQ fell from 4.70% at <1,550 m to 2.97% at >1,850 m and was progressively dampened in older orchards. In mature (>14 years) orchards the altitudinal range narrowed to 3.75–3.13%. The highest single combination was young, low-altitude orchards (A1, <8 years; 4.70%) and the lowest was young, high-altitude orchards (A4, <8 years; 2.97%), showing that the age and altitude effects on MQ are not additive but interact, with youth amplifying the altitudinal contrast (Manzoni et al., 2012; Qiao et al., 2019).

**Figure 5:**
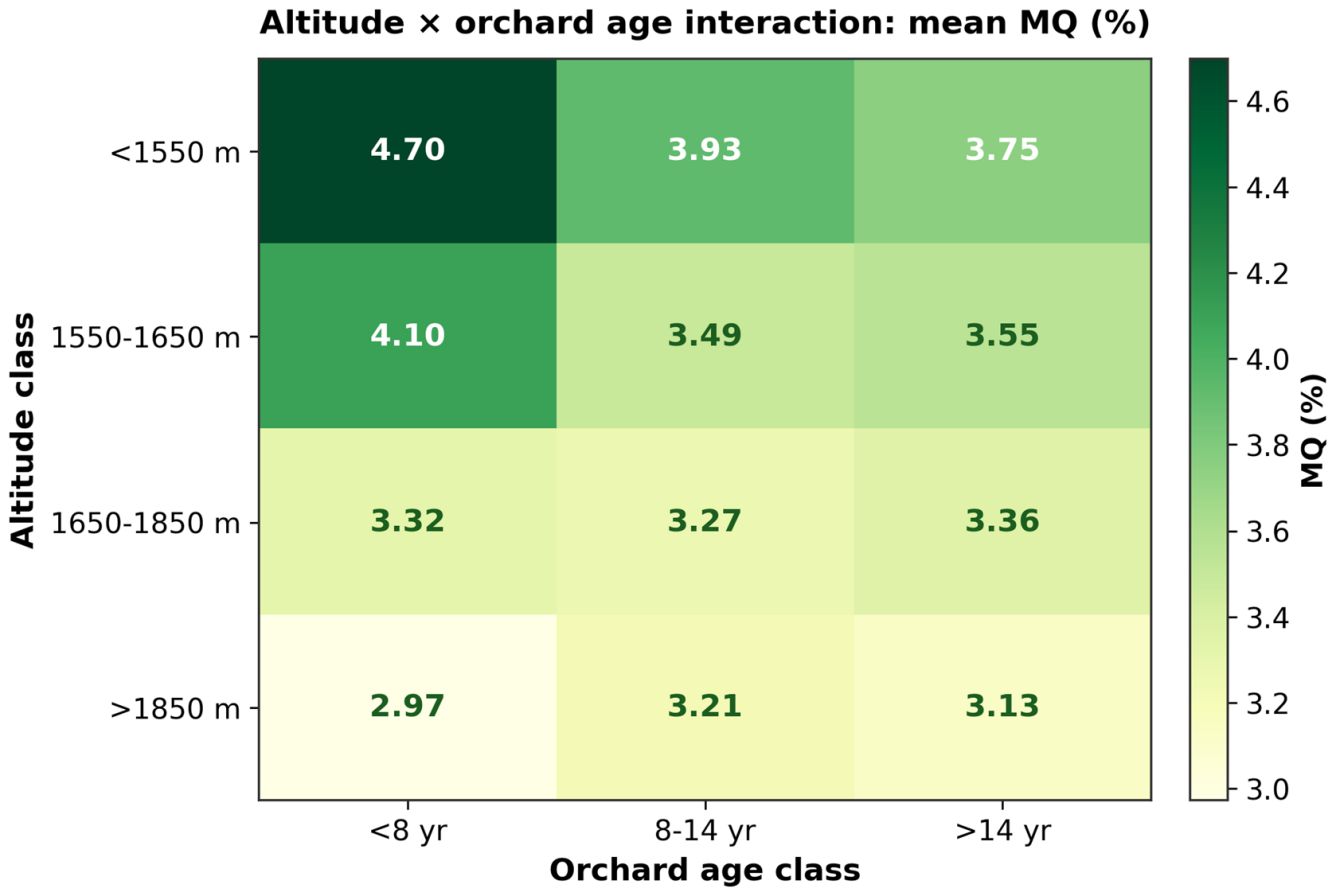
Altitude × orchard age interaction for the microbial quotient (MQ, %). Cell values are means across soil depths and replicates. The heatmap is rendered on the perceptually uniform, colour-blind-safe viridis scale, which retains a monotonic light-to-dark ordering when reproduced in greyscale; lighter cells denote lower MQ and darker cells higher MQ. Numerical values are printed in every cell so that the figure can be read independently of colour.

### 3.7 Correlations among biological indicators

Pearson correlation analysis (Table 6, Figure 6) revealed that MQ was strongly and negatively correlated with TOC (*r* = −0.745, *p* < 0.005) but was essentially uncorrelated with MBC (*r* = −0.082, *p* = 0.328). This contrast is the central diagnostic result: because MQ tracks the substrate denominator far more closely than the biomass numerator, it conveys information that absolute MBC does not. MQ was also negatively correlated with DHA (*r* = −0.343, *p* < 0.005) and alkaline phosphatase (*r* = −0.314, *p* < 0.005) and positively with pH (*r* = 0.318). In contrast, the absolute biological indicators were strongly and positively inter-correlated—MBC with TOC (*r* = 0.671), MBC with DHA (*r* = 0.709) and TOC with DHA (*r* = 0.673)—reflecting their shared increase with altitude and proximity to fresh organic inputs. The decoupling of MQ from MBC, alongside the tight coupling among the absolute pools, accords with the view that the ratio and the biomass measure carry non-redundant information (Wardle, 1992; Anderson, 2003).

**Table 6:** Pearson correlation coefficients among the microbial quotient (MQ) and supporting soil biological indicators (*n* = 144).

Indicator	MQ	MBC	TOC	DHA	P.ase	pH	BD
MQ	1.00						
MBC	−0.08	1.00					
TOC	−0.74***	0.67***	1.00				
DHA	−0.34***	0.71***	0.67***	1.00			
P.ase	−0.31***	0.34***	0.50***	0.38***	1.00		
pH	0.32***	−0.40***	−0.42***	−0.53***	−0.08	1.00	
BD	−0.14	0.13	0.21*	0.33***	0.40***	−0.11	1.00

*p < 0.05; **p < 0.01; ***p < 0.005. BD, bulk density; P.ase, alkaline phosphatase.

**Figure 6:**
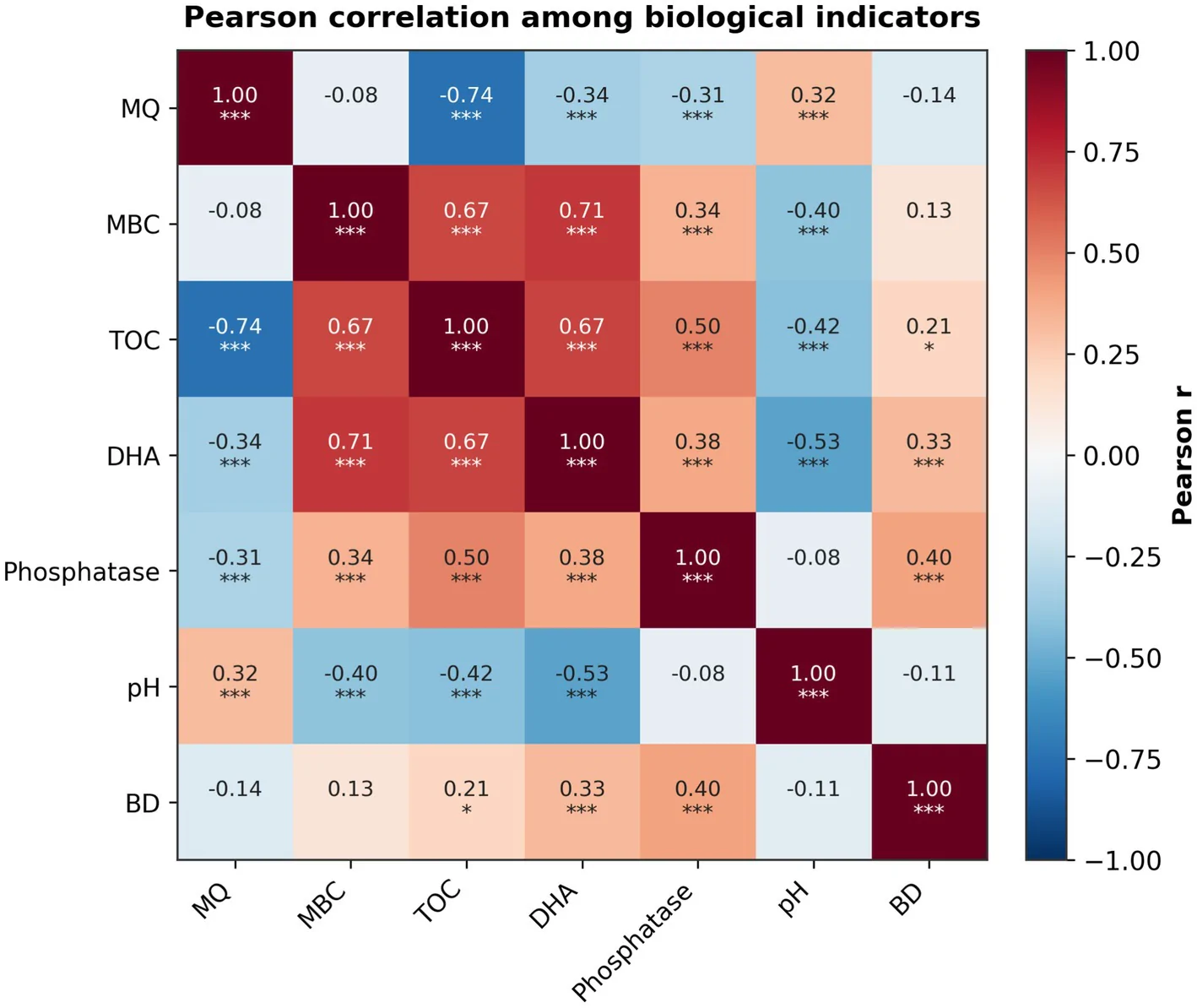
Correlation matrix among the microbial quotient (MQ), microbial biomass carbon (MBC), total organic carbon (TOC), dehydrogenase activity (DHA), alkaline phosphatase, soil pH and bulk density (BD) alkaline phosphatase. Cell colour encodes the Pearson coefficient; asterisks denote significance.

### 3.8 Principal component analysis

Principal component analysis of the seven biological indicators yielded three components with eigenvalues exceeding unity, together explaining 79.1% of the total variance (Table 7, Figure 7). The first component (PC1) accounted for 48.3% of the variance (eigenvalue 3.41) and was a biomass-substrate axis, with strong positive loadings for TOC (0.495), DHA (0.465) and MBC (0.404) and a negative loading for MQ (−0.336); MQ thus loaded opposite to the absolute carbon and activity variables, confirming its inverse relationship with substrate. The second component (PC2, 16.3%) was dominated by bulk density (0.636), alkaline phosphatase (0.538) and pH (0.455), representing a physico-chemical axis. The third component (PC3, 14.5%) was strongly and almost exclusively defined by MQ (0.762), demonstrating that the microbial quotient captures a distinct dimension of biological variation that is not represented by the biomass, activity or physico-chemical axes. The emergence of a dedicated axis for the ratio in ordination space is consistent with multivariate analyses that resolve microbial ratios separately from absolute carbon pools (Bowles et al., 2014; Raiesi and Beheshti, 2015).

**Table 7:** Principal component loadings of soil biological indicators for the first three components (eigenvalue > 1).

Indicator	PC1	PC2	PC3
MQ	−0.336	−0.077	0.760
MBC	0.404	−0.267	0.450
TOC	0.495	−0.044	−0.262
DHA	0.465	−0.140	0.260
Alkaline phosphatase	0.330	0.538	0.050
pH	−0.326	0.455	−0.018
BD	0.220	0.636	0.290
Eigenvalue	3.40	1.15	1.02
Variance explained (%)	48.31	16.31	14.45
Cumulative variance (%)	48.31	64.62	79.07

Loadings with the largest absolute values on each component are those most strongly associated with that axis.

**Figure 7:**
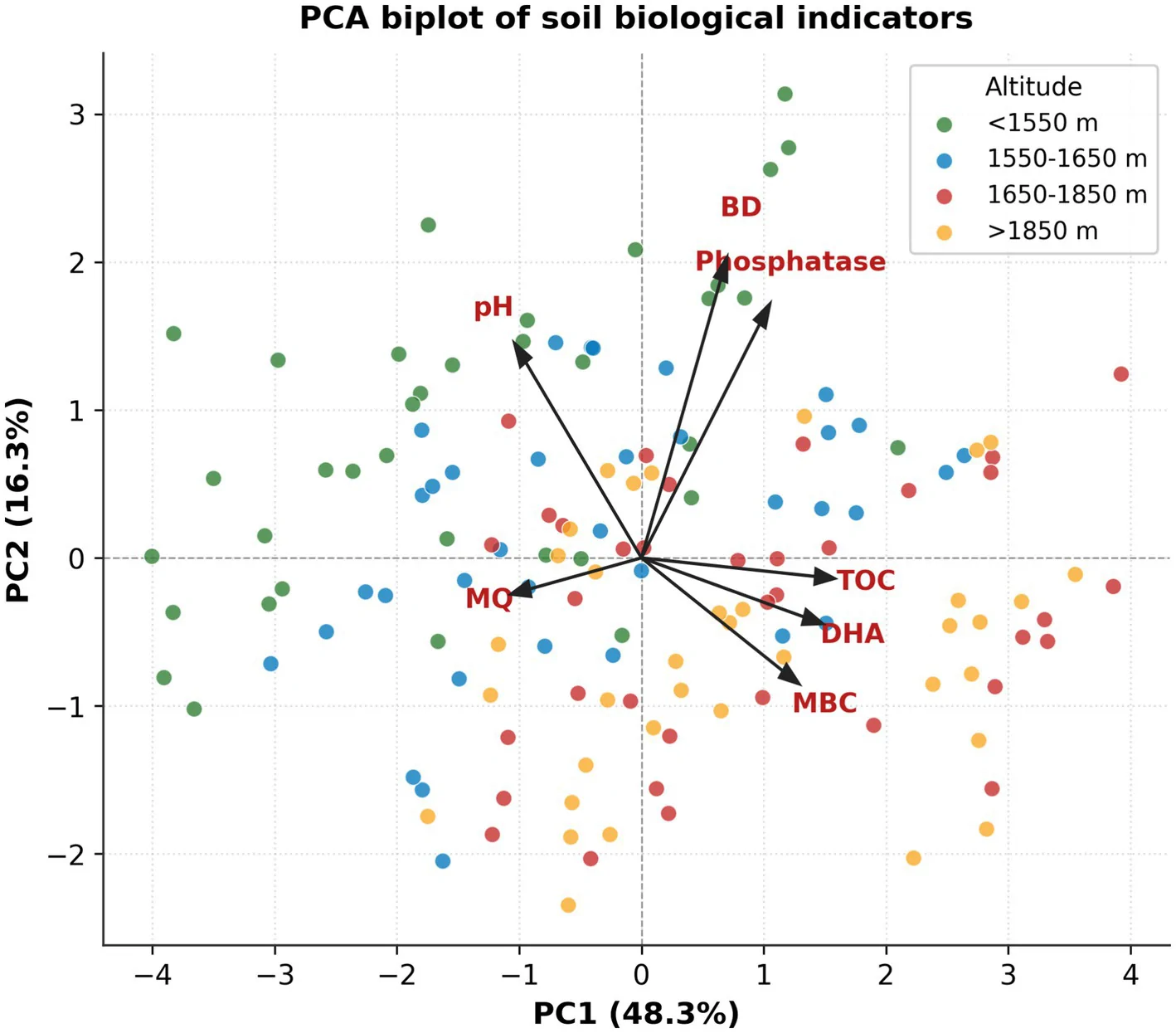
Principal component analysis biplot of soil biological indicators. Sample scores are coloured by altitude class; vectors show indicator loadings on *PC1* and *PC2*. MQ projects opposite to the absolute carbon and activity vectors.

### 3.9 Regression of MQ on biological indicators

Regression analysis quantified the dependence of MQ on each biological indicator. TOC was by far the most influential explanatory variable, accounting alone for 55.6% of the variation in MQ (*R*^2^ = 0.556, slope = −0.09397, *p* < 0.005), the negative slope indicating dilution of the microbial fraction as the substrate pool enlarges. MQ regressed weakly but significantly negatively on DHA (*R*^2^ = 0.118, *p* < 0.005) and alkaline phosphatase (*R*^2^ = 0.099, *p* < 0.005). In contrast, the regression of MQ on MBC was not significant (*R*^2^ = 0.007, *p* = 0.328), reinforcing that variation in MQ is governed by the organic carbon denominator rather than by microbial biomass abundance. The pre-eminence of TOC as the controlling variable agrees with regression-based assessments in which substrate quantity dominates the microbial quotient (Insam and Domsch, 1988; Tashi et al., 2016).

## 4 Discussion

### 4.1 What the microbial quotient measures in this dataset

The central contribution of this study is the demonstration that the microbial quotient behaves as a distinct, substrate-normalised indicator of soil biological status that frequently opposes the trends shown by absolute microbial biomass and organic carbon. Across the Himalayan apple orchards studied, MQ averaged 3.57% and spanned 2.06–8.28%, within the 1–5% band reported for productive agricultural and orchard soils and below the higher values typical of undisturbed forest and grassland (Anderson and Domsch, 1989; Sparling, 1992).

It is important to state at the outset how far the present data can be interpreted. MQ is a stoichiometric ratio between two measured carbon pools. It is widely used as a proxy for microbial carbon-use efficiency, but it is not a direct measure of that quantity: carbon-use efficiency is properly determined by substrate-labelling, calorimetric or isotopic approaches, none of which was applied here. Neither microbial community composition, necromass content nor the metabolic pathways involved were measured in this study. Accordingly, the mechanistic accounts offered below are presented as explanations consistent with the observed patterns and with the published literature, not as mechanisms demonstrated by these data. What the study establishes empirically is the pattern of MQ across altitude, age and depth, its statistical dependence on TOC rather than MBC and its statistical independence from the conventional abundance indicators.

This matters because soil-health research on Himalayan orchards has largely reported absolute carbon and biomass pools, which cannot by themselves indicate how large the living fraction is relative to the substrate that sustains it (Dad and Abdollahi, 2021; Khenrab et al., 2025; Dutta and Pattanayak, 2024). The moderate coefficient of variation (28%) shows that MQ was responsive enough to discriminate site conditions while remaining stable and interpretable and the gradient-structured grand mean of 3.57% furnishes the first quantitative baseline for biological monitoring of these orchards (Anderson, 2003; Saljnikov et al., 2022).

### 4.2 Altitudinal decline

The significant decline of MQ with altitude, from 4.13% at the lowest sites to 3.11% at the highest, is the inverse of the increase in absolute MBC and TOC that characterises these and other mountain soils (Margesin et al., 2009; Dutta and Pattanayak, 2024). Two mechanisms could plausibly produce this divergence. Decreasing temperature may slow decomposition more than carbon input, so that organic carbon accumulates and dilutes the microbial fraction in the MQ denominator; and lower temperatures may reduce carbon-use efficiency through higher maintenance respiration, so that less assimilated carbon is retained as biomass (Frey et al., 2013; Manzoni et al., 2012). The present data can discriminate between these only indirectly. The strong negative MQ-TOC correlation (*r* = −0.75) together with the weak, non-significant MQ-MBC relationship is consistent with the first, substrate-dilution explanation being the dominant one, since MQ varies with the denominator while the numerator remains comparatively invariant; the second, efficiency-based explanation cannot be evaluated without direct carbon-use-efficiency measurements. A decline in microbial metabolic efficiency with elevation despite rising absolute carbon pools has likewise been reported from the Tibetan Plateau (Feng et al., 2021).

### 4.3 The subsurface maximum

The depth behaviour of MQ is the most diagnostically valuable result. Whereas MBC, TOC and enzyme activities all declined monotonically from the surface downward, MQ followed the opposite pattern, rising from 2.82% at the surface to a subsurface maximum of 4.14% at 25–50 cm—an increase of nearly 47% that is highly significant in the ANOVA and consistent across altitude classes (Fierer et al., 2009; Loeppmann et al., 2016).

This decoupling indicates that the surface horizon, though richest in carbon and biomass, holds the smallest proportion of its carbon in living biomass. A plausible reading, consistent with profile-resolved studies elsewhere, is that its large litter- and pruning-derived carbon pool includes a substantial partly recalcitrant fraction that dilutes the active biomass (Paul, 2016; Rasmussen et al., 2018), whereas in the subsurface, where carbon is scarcer and dominated by root-derived, microbially reworked material, a greater proportion is associated with living biomass. Whether microbial necromass contributes to this subsurface pattern, as the anabolic model of soil carbon formation would predict, cannot be established here because necromass biomarkers such as amino sugars were not determined (Kallenbach et al., 2016; Liang et al., 2017). Independently of mechanism, the result carries a clear practical implication: surface-only sampling would misrepresent the depth distribution of the microbial fraction and would in this dataset have understated MQ by roughly one-third.

### 4.4 Orchard age and its interaction with altitude

The orchard age effect, though significant in the ANOVA, was modest and its pairwise contrasts did not survive Tukey correction, indicating a weak directional trend that should not be over-interpreted. The decline from 3.77% in young orchards to about 3.45% in mature ones is in the direction expected as a perennial system accumulates stabilised carbon (Six et al., 2006; Jakhro and Li, 2025). Long-term fertiliser inputs may also contribute, since sustained mineral nitrogen application in orchard agrosystems alters the partitioning of nitrogen between microbial and abiotic pools and thereby the resource context in which the biomass operates (Zheng et al., 2024). The significant altitude × age interaction refines the picture: the altitudinal decline was steepest in young orchards, so the youngest, lowest-altitude sites attained the highest MQ (4.70%) and young high-altitude sites the lowest (2.97%), with the contrast converging as orchards mature (Zhang et al., 2019; Qiao et al., 2019).

### 4.5 Enzyme activities and ecological stoichiometry

The supporting enzymes place these patterns in a resource context. DHA and alkaline phosphatase both correlated positively with MBC and TOC and negatively with MQ (Moscatelli et al., 2018; Sinsabaugh et al., 2008). The negative MQ-enzyme correlations follow directly from the substrate-dilution framework: high enzymatic throughput accompanies large substrate pools, yet large substrate pools lower the proportion of carbon held as biomass—a pattern consistent with eco-enzymatic theory, in which enzyme investment tracks resource availability (German et al., 2011; Sinsabaugh et al., 2016).

These enzyme responses are best interpreted within the broader framework of soil ecological stoichiometry. The C:N:P ratio of the soil and its imbalance relative to microbial demand, is a fundamental regulator of microbial biomass, nutrient cycling and biological efficiency and it constrains how much of the carbon a community processes can be retained as biomass rather than respired (Liu et al., 2023; Cui et al., 2018). Viewed stoichiometrically, the altitudinal decline in MQ reported here is not simply a carbon phenomenon: as organic carbon accumulates in cold, high-elevation soils, the soil C:N and C:P ratios widen and a community facing an increasingly carbon-rich but nutrient-poor substrate must invest proportionally more in nutrient-acquiring enzymes while retaining proportionally less carbon as biomass. The elevated alkaline phosphatase activity that accompanied high TOC and low MQ in this dataset is exactly what such widening C:P stoichiometry would predict and it offers a coherent theoretical framework for interpreting MQ that extends beyond simple carbon dynamics. Direct measurement of soil and microbial biomass C:N:P, together with the full suite of C-, N- and P-acquiring enzymes, is the natural next step in testing this interpretation and would allow the enzyme vector approach to be used to identify whether these communities are carbon- or nutrient-limited.

### 4.6 Coupled nutrient cycling

Because the present study measured carbon-cycle indicators alongside only one nutrient-acquiring enzyme, the coupling between MQ and nutrient cycling deserves explicit comment. Dehydrogenase and alkaline phosphatase index, respectively, oxidative metabolism and organic phosphorus mineralisation and both are microbially mediated steps in the wider nutrient cycle rather than in carbon turnover alone. Microbial communities regulate nitrogen and phosphorus transformations through redox-sensitive and enzyme-mediated pathways whose rates are set jointly by organic matter quality, electron availability and community composition, so that carbon and nutrient cycles are functionally inseparable in soils (Zeng et al., 2024; Chen et al., 2018). The subsurface MQ maximum reported here is therefore likely to have nutrient-cycling as well as carbon-cycling significance, since a larger living biomass fraction per unit carbon implies a larger immobilisation-mineralisation turnover capacity in exactly the layers where apple roots are most active. Testing this requires measurement of nitrogen and phosphorus pools and of the corresponding N- and P-cycling enzymes and functional genes alongside MQ, which was beyond the scope of the present survey.

### 4.7 Multivariate structure and pH

The PCA reinforced that MQ measures something the absolute indicators do not. The first axis was a biomass-substrate gradient on which MBC, TOC and DHA loaded positively and MQ negatively, while the third axis was defined almost entirely by MQ. This dedicated dimension shows that the ratio is statistically orthogonal to abundance and substrate quantity, so MQ adds genuinely independent information: an indicator battery dominated by abundance measures would be largely redundant, whereas MQ captures a dimension they miss (Trivedi et al., 2016; Marinari et al., 2022).

Soil pH was a secondary but coherent modulator, with a positive MQ-pH correlation and a negative pH-enzyme relationship. The mildly acidic to neutral reaction of these soils is generally associated with bacterial-dominated communities and the positive MQ-pH association is in the direction reported where carbon-use efficiency declines under increasing acidity and fungal dominance, although community composition was not determined here (Rousk et al., 2010; Malik et al., 2018). Because high-altitude sites were also more acidic, the pH effect is confounded with and would reinforce, the temperature- and substrate-related altitudinal decline (Margesin et al., 2009; Delgado-Baquerizo et al., 2016).

### 4.8 Implications for carbon cycling under climate change and for orchard management

These patterns carry implications for carbon cycling under a warming climate. The high-altitude orchards that store the most organic carbon also hold the smallest proportion of that carbon in living biomass and their stocks are currently protected in part by cold-limited metabolism (Frey et al., 2013; Wieder et al., 2013). If warming raises microbial efficiency and accelerates turnover, as models and translocation experiments suggest, these soils could shift from sinks toward sources; under the anabolic view of soil carbon formation, a depressed MQ would additionally signal a thinner reserve of microbially derived, mineral-associated carbon for the future (Nottingham et al., 2019; Zhou et al., 2020; Crowther et al., 2016). This projection is a hypothesis generated by, not tested in, the present dataset.

Read as a practical monitoring tool, the results translate into four specific management recommendations for climate adaptation in the Jammu apple belt. First, high-altitude and mature orchards, where MQ is intrinsically lowest, should be prioritised for organic carbon inputs in labile form—farmyard manure, orchard-floor mulching with pruning residues and legume-based cover cropping in the alleys—since these supply the fresh substrate with which a larger biomass fraction is associated. Second, because MQ responds to the ratio rather than the amount of carbon, sequestration-oriented practices should be evaluated against a biological metric and not against TOC gain alone; an increase in TOC accompanied by a fall in MQ would indicate carbon accruing in a form the community is not processing. Third, monitoring should be depth-resolved to at least 50 cm, since the subsurface maximum documented here is invisible to conventional 0–15 or 0–25 cm sampling. Fourth, given the positive MQ-pH association and the greater acidity of high-elevation sites, pH management through periodic liming to the near-neutral optimum warrants evaluation as a low-cost intervention in the upper altitudinal classes. Each recommendation follows from the observed gradients and each is a testable proposition rather than a validated prescription (Doran and Zeiss, 2000; Lori et al., 2017; Jakhro et al., 2025b).

### 4.9 Limitations and future perspectives

Several limitations temper these conclusions and define the agenda for further work. The analysis rests on a single sampling campaign and so captures spatial but not temporal variation in MQ, which is known to fluctuate seasonally (Schimel, 2018; Yang et al., 2023). The enzyme suite does not resolve community composition. Most importantly, microbial carbon-use efficiency, necromass and metabolic pathways were not measured, so the substrate-dilution and efficiency interpretations advanced above remain hypotheses, however consistent they are with the MQ-TOC relationship (Nannipieri et al., 2017; Sinsabaugh et al., 2016). The study should therefore be read as establishing a baseline: it defines what MQ does across these gradients and identifies the variable it tracks, leaving the underlying biology to be resolved.

Future work should move beyond generic calls for molecular profiling toward a defined multi-omic and stoichiometric programme. Shotgun metagenomics would establish the taxonomic composition and the functional gene inventory—carbohydrate-active enzymes, nitrogen- and phosphorus-cycling genes—underlying the MQ gradients reported here, while metatranscriptomics would distinguish genes that are merely present from those actively expressed at each depth and elevation, which is essential if the subsurface maximum is to be interpreted as active rather than residual biomass. Metabolomics of soil solution and of microbial extracts would characterise the substrate quality that the MQ denominator conceals and would test directly whether subsurface carbon is the microbially reworked material the present data imply. Microbial ecological network analysis across the altitudinal gradient would reveal whether the low-MQ, high-elevation soils also support less connected or less stable communities, since network topology and community stability respond to nutrient status in ways that biomass measurements alone cannot detect; the value of coupling network and stability metrics to nutrient gradients has recently been demonstrated in other environmental systems (Hou et al., 2026; Yang et al., 2023). Amplicon-based microbiome surveys of the bacterial and fungal communities would allow the fungal:bacterial ratio to be tested as a co-predictor of MQ. Alongside these, direct isotopic determination of carbon-use efficiency, amino-sugar quantification of necromass, full soil and microbial biomass C:N:P stoichiometry and seasonal repeat sampling are required before the descriptive benchmarks reported here can be developed into validated, season-specific MQ thresholds for the Himalayan apple belt (Liang et al., 2017; Liu et al., 2023; Marinari et al., 2022).

## 5 Conclusion

This study provides the first systematic assessment of the microbial quotient across the altitude, orchard age and soil depth gradients that structure Himalayan apple production systems in the Jammu region. MQ averaged 3.57% and declined significantly with altitude (from 4.13% at <1,550 m to 3.11% at >1,850 m) and, more weakly, with orchard age, while exhibiting a distinctive subsurface maximum (4.14% at 25–50 cm) that opposed the surface-concentrated distribution of absolute microbial biomass and organic carbon. The defining result is that MQ was strongly and negatively related to total organic carbon (*r* = −0.75; *R*^2^ = 0.56) yet effectively decoupled from microbial biomass carbon and that principal component analysis resolved a dedicated MQ axis of variation. Together these findings establish that MQ conveys information about the substrate-normalised size of the living biomass pool that conventional biomass and carbon measures cannot supply.

These results should be understood as baseline observations. The study characterises the behaviour of MQ across three environmental gradients and identifies TOC as the variable that governs it, but because microbial carbon-use efficiency, community composition, necromass and functional gene expression were not measured, the efficiency-based mechanisms proposed to explain these gradients remain to be validated. Future studies integrating microbial sequencing, functional gene analysis and ecological stoichiometry are required to test whether the patterns documented here do in fact reflect variation in microbial carbon-use efficiency and to convert the descriptive benchmarks into mechanistic understanding.

For sustainable orchard management, MQ nevertheless offers a single, inexpensive, substrate-normalised index of biological soil status that is comparable across sites of contrasting organic carbon content. The subsurface maximum argues for depth-resolved biological monitoring to at least 50 cm and the altitude × age interaction indicates that practices supplying fresh, labile carbon—organic mulching, cover cropping and balanced nutrition—should be prioritised in mature and high-altitude orchards where MQ is intrinsically lowest. Because the high-altitude orchards that store the most carbon also hold the least of it as living biomass, their carbon stocks may be especially vulnerable to climate warming and MQ provides a practical early-warning metric for this risk.

Three directions for future research follow directly from these limitations. First, the present spatial assessment should be extended to multiple seasons, since a single post-monsoon campaign cannot capture the seasonal amplitude of MQ and season-specific thresholds will be needed before MQ can serve as an operational monitoring benchmark. Second, MQ should be coupled with direct measurements of microbial carbon-use efficiency, community composition and necromass, so that the mechanistic interpretation can be tested rather than inferred. Third and most important for practice, the responsiveness of MQ to defined management interventions should be established through controlled trials—mulching, cover cropping, organic amendment and liming applied at known rates with MQ monitored over successive seasons—so that the magnitude and time-scale of MQ response to management is quantified and biological benchmarks for mountain apple orchards can be refined into operational thresholds.

## Data Availability

The original contributions presented in the study are included in the article; further inquiries can be directed to the corresponding authors.
